# Effect of Vaccination on Transmission of SARS-CoV-2

**DOI:** 10.1056/NEJMc2106757

**Published:** 2021-09-08

**Authors:** Anoop S.V. Shah, Paul M. McKeigue, David A. McAllister, And Others. A complete list of authors is available with the full text of this letter at NEJM.org., Anoop S.V. Shah, Ciara Gribben, Jennifer Bishop, Peter Hanlon, David Caldwell, Rachael Wood, Martin Reid, Jim McMenamin, David Goldberg, Diane Stockton, Sharon Hutchinson, Chris Robertson, Paul M. McKeigue, Helen M. Colhoun, David A. McAllister

**Affiliations:** London School of Hygiene and Tropical Medicine London, United Kingdom; University of Edinburgh Edinburgh, United Kingdom; University of Glasgow Glasgow, United Kingdom; London School of Hygiene and Tropical Medicine London, United Kingdom; Public Health Scotland Edinburgh, United Kingdom; Public Health Scotland Edinburgh, United Kingdom; University of Glasgow Glasgow, United Kingdom; Public Health Scotland Edinburgh, United Kingdom; University of Edinburgh Edinburgh, United Kingdom; Public Health Scotland Edinburgh, United Kingdom; Public Health Scotland Edinburgh, United Kingdom; Public Health Scotland Edinburgh, United Kingdom; Public Health Scotland Edinburgh, United Kingdom; Glasgow Caledonian University Glasgow, United Kingdom; University of StrathclydeGlasgow, United Kingdom; University of Edinburgh Edinburgh, United Kingdom; University of Edinburgh Edinburgh, United Kingdom; University of Glasgow Glasgow, United Kingdom

To the Editor:

To determine whether vaccinating individuals for SARS-CoV-2 protects those around them, we examined whether vaccinating healthcare workers in Scotland (in global terms among the earliest groups to be vaccinated) was associated with a lower risk of COVID-19 among members of their households.

We studied 194,362 household members (92,470 households, 2-14 individuals per household) of 144,525 healthcare workers (mean ages 31 and 44 years respectively, both >96% white) employed throughout March to November 2020. 113,253 (78.3%) healthcare workers received at least one dose of the BNT162b2 mRNA or ChAdOx1 nCoV-19 vaccine and 36,227 (25.1%) received a second dose.

The primary outcome was any incident documented case of COVID-19 from 8^th^ December 2020 to 3^rd^ March 2021. We also report results for COVID associated hospitalization. The primary comparison was between the unvaccinated period and 14-days after the healthcare worker received their first dose. No adjustment for multiplicity was made. Events after any household member was themself vaccinated were censored. Detailed methods and results, strengths and limitations, and our pre-specified protocol are available in the Supplement. This project was approved by the Public Benefit and Privacy Panel (2021-0013) and the Scientific Officer of the West of Scotland Research Ethics Committee confirmed in writing that formal ethics review was not required.

Cases of COVID-19 were less common among household members of vaccinated healthcare workers (rate per 100 person-years 9.40 and 5.93 pre-first dose and 14-days post first dose respectively). After the healthcare worker’s second dose the rate in household members was lower still (2.98 per 100 person-years). These differences persisted after fitting extended Cox models adjusted for calendar time, region, age, sex, occupational and socio-economic factors, and underlying conditions; relative to the period before each healthcare worker was vaccinated the hazard ratios were 0.70 [95% confidence interval (CI) 0.63-0.78] and 0.46 [95%CI 0.30-0.70] for the periods 14-days after the first and second doses respectively (Table and Supplement). Not all cases of COVID-19 in household members are via the healthcare worker route so the effect of vaccination may be larger.^1^ For example, if half of all cases in household members were via the healthcare route, a 60% fall in cases obtained via this route would be needed to create the association we observed (Supplement). Vaccination was associated with reduction in both cases and hospitalisation of COVID-19 in healthcare workers.

Vaccination reduces asymptomatic infection with SARS-COV-2,^2,3^ making it plausible that vaccination reduces transmission, but this has not been examined in clinical trials or observational studies.^4,5^ We provide empirical evidence suggesting that vaccination may reduce transmission by showing that vaccinating healthcare workers is associated with a fall in documented cases of COVID-19 among members of their households. This finding is reassuring for healthcare workers and their families.

## Supplementary Material

Appendix

## Figures and Tables

**Table:** Effect of vaccination in healthcare workers on documented COVID-19 cases and hospitalizations in healthcare workers and their households: unvaccinated period versus period from 14-days post first dose

	Healthcare workers				Household members			
	Cases^a^		Hospitalizations		Cases^a^		Hospitalizations	
	Unvaccinated period	Post first dose	Unvaccinated period	Post first dose	Unvaccinated period	Post first dose	Unvaccinated	Post first dose
n	144525	109074	144525	111081	194362	148366	194362	149689
Events	3191	1152	158	19	2037	1086	111	64
Mean person time (days)	40	45	41	45	41	45	41	45
Rate per 100 person years	20.13	8.51	0.97	0.14	9.40	5.93	0.51	0.35
Models (Hazard ratios)								
Unadjusted	0.51 (0.48-0.55)		0.16 (0.10-0.27)		0.74 (0.67-0.82)		0.83 (0.58-1.17)	
Model 1	0.52 (0.49-0.56)		0.16 (0.10-0.27)		0.73 (0.66-0.81)		0.81 (0.57-1.15)	
Model 2	0.55 (0.51-0.59)		0.17 (0.10-0.29)		0.75 (0.68-0.83)		0.86 (0.61-1.23)	
Model 3	0.45 (0.42-0.49)		0.15 (0.09-0.26)		0.71 (0.63-0.78)		0.77 (0.53-1.10)	
Model 4^b^	0.45 (0.42-0.49)		0.16 (0.09-0.27)		0.70 (0.63-0.78)		0.77 (0.53-1.10)	

Results are shown for Cox regression models stratified by health board area, with the timescale being calendar time and the exposure treated as a time-varying covariate. Models were adjusted sequentially for age (using a penalized spline to allow for non-linearity) and sex (model 1); Scottish Index of Multiple Deprivation (an area-based measure of socio-economic deprivation – SIMD) and ethnicity (model 2), healthcare worker role (patient facing, non-patient facing, undetermined), occupation and part-time status (model 3); and comorbidity (as both a comorbidity count and the presence/absence of type 2 diabetes) (model 4).

a Cases were defined on polymerase chain reaction testing for COVID-19 (estimated at 90% sensitivity and 99% specificity in Scotland – Wellcome Open Res 2020;5:254.doi:10.12688/wellcomeopenres.16342.1)

b In sensitivity analyses testing the robustness of the findings to the proportional hazards assumption all covariates were instead included as stratifying variables in the extended Cox models (age was categorised in ten-year bands with the remaining covariates treated as unordered categorical variables). In these models the hazard ratios were 0.44 (95% CI 0.40-0.47) and 0.68 (95% CI 0.62-0.75) for cases in healthcare workers and cases in household members respectively.
