# Supplementary material for: Effect of Vaccination on Transmission of SARS-CoV-2
Source: N Engl J Med. Author manuscript; Available in PMC 2021 Oct 28. (PMC8451182; doi:10.1056/NEJMc2106757)
Supplement: Appendix [file EMS134656-supplement-Appendix.pdf]

TABLE OF CONTENTS

1.1 Supplementary methods **2**
1.1.1 Population, data sources and record linkage 2
1.1.2 Outcomes and exposures 2
1.1.3 Covariates 2
1.1.4 Statistical analysis 3

1.2 Supplementary text **4**
1.2.1 Supplement text 1: Description and sources of national datasets used for individual record linkage 4
1.2.2 Supplement text 2: Strengths and limitations 7

1.3 Supplementary figures **8**
1.3.1 Supplement figure 1: Overview of record linkage 8
1.3.2 Supplement figure 2: Participant inclusion and exclusion for healthcare workers and their household members 9
1.3.3 Supplement figure 3: Heatmap indicating date of first and second dose of vaccination among healthcare workers. Lighter color indicates higher number receiving vaccines on those dates. 10

1.4 Supplement tables **11**
1.4.1 Table 1 Baseline characteristics of vaccinated and unvaccinated and their household members by month of first dose in healthcare workers. 11
1.4.2 Table 2: Estimation of the number of cases prevented in household members by vaccinating healthcare workers as a fraction of the number of cases preventable via interrupting this route of transmission (ie those cases caused by sharing a household with a healthcare worker) 14
1.4.3 Table 3: Effect of vaccination in healthcare workers on documented COVID-19 in healthcare workers and their household at multiple time periods following the first dose 15
1.4.4 Table 4: Effect of second dose vaccination in healthcare workers on documented COVID-19 cases and hospitalizations in healthcare workers and their households: unvaccinated period versus periods from 14-days post second dose 16

1.5 References **17**

1.6 Study protocol **18**

1.1 Supplementary methods

1.1.1 Population, data sources and record linkage

Healthcare workers were included if they were employed by the National Health Service (NHS) in Scotland on or before the 1st of March 2020 (the first positive reported case of COVID-19 in Scotland) and still employed by the NHS on the 1st of November 2020. Healthcare workers with a positive COVID-19 polymerase chain reaction (PCR) test before the 8th December 2020 (the date when the vaccination programme was initiated) were excluded from the analysis. Data on healthcare workers and their household members were extracted and linked to multiple national datasets as previously described. In brief the Community Health Index (CHI) database, a registry of all patients registered to receive care from the NHS in Scotland (close to the complete population) was linked to the Scottish Workforce Information Standard System (SWISS) and General Practitioner Contractor Database (GPCD) databases. The CHI database was used to identify all individuals who were not themselves healthcare workers but shared a household with a healthcare worker. The healthcare worker and household data were then linked to multiple national databases including virology testing for SARS-CoV-2, general hospitalisation data, community prescribing, critical care admissions and the national register for deaths (Supplementary text 1 and Supplementary figure 1).14 The healthcare worker cohort was restricted to the working-age population (18-65 years of age). The household member cohort included all ages but was restricted to households with no more than one healthcare worker (4% of healthcare workers lived in multiple healthcare worker households) (Supplementary figure 2).

1.1.2 Outcomes and exposures

Outcomes were restricted to the time period from the 8th December 2020 to 3rd March 2021. The pre-specified primary outcome was any positive PCR test for SARS-CoV-2 (hereafter documented COVID-19). The secondary outcome was death or hospitalisation with COVID-19 defined hospitalisations/deaths where the recorded cause was COVID-19 (ICD-10 U07.1,U07.2 or U07.5) or any hospitalisation/death occurring within 28 days of a first positive test or having first tested positive while in hospital. Where hospitalisation or death occurred without any positive test (only five and three events in healthcare workers and household members respectively), the event date was presumed to have occurred 14 days prior to the date of hospitalisation and/or death. The exposure was defined when a healthcare worker received the first dose of the BNT162b2 mRNA or ChAdOx1 nCoV-19 vaccine (the only two vaccines approved for clinical use at that time in the UK). A second dose of these vaccines was studied as a separate exposure. Supplementary figure 3.

1.1.3 Covariates

Covariates were obtained from nationwide databases as previously described.[14] Health board area was included as a stratifying variable, and other variables identified as potential risk factors for COVID-19 hospitalization were included as covariates in regression models; age, sex, Scottish Index of Multiple Deprivation (an area-based measure of socio-economic deprivation - SIMD), ethnicity, comorbidity (as both a comorbidity count and the presence/absence of type 2 diabetes), healthcare worker role (patient facing, non-patient facing or undetermined), occupation and part-time status.

1.1.4 Statistical analysis

The statistical analysis was pre-specified prior to linking the outcome and vaccination data (http://www.encepp.eu/encepp/viewResource.htm?id=39737, https://github.com/dmcalli2/hcw_vax). Person-time at risk was defined as the period from the 8th December 2020 to the date of the target event, death from a non-COVID-19 cause, or the end of follow-up (3rd March 2021), whichever came first. If household members were themselves vaccinated, person-time was censored on the day of their vaccination. Vaccination status was encoded as a time-varying categorical variable with three levels, defined with respect to the date of the first dose – unvaccinated for all days prior, intermediate for days 1 to 13, and post-dose from day 14 onwards. For additional analyses examining the effect of the second dose, we added a further level - 14 days onward from the second dose. Extended Cox regression models were used to estimate hazard ratios (HRs) for the effect of vaccination on both cases and hospitalisation, calculating robust standard errors to allow for clustering due to shared household membership and stratifying on health board area to allow for regional differences in baseline hazard.

Hazard ratios were reported unadjusted and then adjusted sequentially for socio-demographic, occupational and comorbidity covariates. Cox regression, with calendar time as the timescale, eliminates the baseline hazard rate and thus makes it unnecessary to model how the rate of COVID-19 varies over calendar time. The pre-specified primary comparison was the HR for documented cases in the period 14 days onward from the first dose compared to unvaccinated person time (i.e. before day 1 of vaccination). We also report pre-specified secondary analyses for the HRs associated with vaccination status at 7-day intervals from the date of vaccination. To test the robustness of the findings to the proportional hazards assumption, we repeated the primary analysis for household members and healthcare workers treating all of the remaining covariates as stratifying variables rather than including them as covariates (see footnote of Table in main manuscript). Analyses were performed in R Version (3·6·1).

1.2 Supplementary text

1.2.1 Supplement text 1: Description and sources of national datasets used for individual record linkage

1.2.1.1 Scottish hospitalization record from SMR01

Comorbidities were defined from previous defined from the Scottish morbidity record 01 (SMR01) - General/Acute Inpatient & Day Case. SMR01 is an episode-based patient record relating to all inpatients and day cases discharged from non-obstetric and non-psychiatric specialties. A record is generated when a patient completes an episode of inpatient or day case care. Data collected include patient identifiable and demographic details, episode management details and general clinical information. Currently diagnoses are recorded using the ICD-10 classification and operations are recorded using the OPCS-4 classification. Further information on the national dataset and variables contained is available at https://www.ndc.scot.nhs.uk/Data-Dictionary/SMR-Datasets//Episode-Management/SMR-Record-Type/

Reports of the regular assessments of the quality of clinical coding are available at: https://beta.isdscotland.org/products-and-services/data-quality-assurance/dqa-assessments/

1.2.1.2 National Records of Scotland (NRS)

The NRS covers all deaths in Scotland with approximately 55,000 deaths registered annually. The National Records of Scotland Death Records are linked with the NHS Scotland Scottish Morbidity Database which links together NHS Scotland inpatient, mental health and cancer registry datasets with the NRS Death Records. NRS records death status, cause of death and date of death.

Further information of the NRS death registry is available at https://www.ndc.scot.nhs.uk/National-Datasets/data.asp?SubID=13

1.2.1.3 Prescribing Information System (PIS)

The Prescribing Information System (PIS) is the definitive data source for all prescribing relating to all medicines and their costs that are prescribed and dispensed in the community in Scotland. The information is supplied by Practitioner & Counter Fraud Services Division (P&CFS) who is responsible for the processing and pricing of all prescriptions dispensed in Scotland. Primary care physicians write the vast majority of these prescriptions, with the remainder written by other authorised prescribers such as nurses and dentists. Also included in the dataset are prescriptions written in hospitals that are dispensed in the community. Note that prescriptions dispensed within hospitals are not included.

Further information on the Prescribing Information System operational in Scotland is available at https://www.ndc.scot.nhs.uk/National-Datasets/data.asp?SubID=9

1.2.1.4 National microbiology register (Electronic Communication of Surveillance in Scotland [ECOSS])

The Scottish microbiology surveillance registry, or '*Electronic Communication of Surveillance in Scotland*' (ECOSS) as it is termed by NHS National Services Scotland, was used in the present study to provide individual patient-level data on SARS-Cov-2 testing and results. ECOSS is part of NHS Scotland's Infection Intelligence Platform (IIP),[2,3] which was set-up in response to the UK's antimicrobial resistance (AMR) strategy (2013-2018) with the aim of providing "*better access to and use of surveillance data*".[4]

Data were first collected and recorded within ECOSS in 2007. The dataset is maintained by NHS National Services Scotland on behalf of Health Protection Scotland. ECOSS is updated monthly and, as of 2017, it contained approximately 29 million records of positive microbiology laboratory specimens from across Scotland.[2] It provides data for numerous national clinical and research activities, audit projects and Scottish Government reports, including: the identification of cases of severe infectious disease, infectious disease outbreaks and the evaluation of longer term trends in the incidence of laboratory-reported infections; surveillance of episodes of Clostridium difficile infections, Escherichia coli bacteremia, Staphylococcus aureus bacteremia and surgical site infections.[3] NHS National Services Scotland monitors the completeness and accuracy of ECOSS data through its 'Data Monitoring and Support Service'.[2] Further, NHS National Services Scotland routinely informs data users of any problems affecting the accuracy or assurance of these data.

More information on the ECOSS data system is available at https://www.hps.scot.nhs.uk/data/

1.2.1.5 Scottish Workforce Information Standard System (SWISS)

The Scottish Workforce Information Standard System (SWISS) is a national human resources database held by NHS Education Scotland which contains data on all directly employed staff (ie not contracted staff such as general practitioners except where they are also directly employed in some other role) working in the NHS in Scotland. It includes data for territorial health boards, and boards providing a national service. It records the job title using a nationally agreed standard as well as, for medical and dental staff the medical specialty. It also includes data on occupation grade, part-time/whole time status, and the designated service area.

More information on the SWISS database is available at https://turasdata.nes.nhs.scot/media/2prjxbg4/2020-06-02-workforce-report.pdf.

1.2.1.6 General Practitioner Contractor Database (GPCD)

The General Practitioner Contractor Database (GPCD) includes all contracted general practitioners working in Scotland. This includes GP partners who are independent contractors, and salaried GPs, but not locum GPs. GOs in training grades are employed centrally and so are included in SWISS.

More information on the GPCD database is available at https://www.isdscotland.org/Health-Topics/General-Practice/Workforce-and-Practice-Populations/.

1.2.1.7 Rapid preliminary inpatient data (RAPID)

The RAPID database has been operational since to 2001 to monitor and predict emergency admissions and bed occupancy across National Health Service Boards in Scotland. Data from this database has already been used to provide information to NHS boards, healthcare workers and the public on the direct and indirect effects of the COVID-19 pandemic. These dashboards can be found at: https://publichealthscotland.scot/our-areas-of-work/sharing-our-data-and-intelligence/coronavirus-covid-19-data/

In 2015 the data collection was expanded to include data items on individual patient level data on age, sex, times of admission and discharge, ward significant facility, diagnosis and operation codes and information on patient discharge.

Further information on the RAPID databases can be found here:
https://www.ndc.scot.nhs.uk/National-Datasets/data.asp?ID=1&SubID=37

1.2.2 Supplement text 2: Strengths and limitations

Our existing cohort of healthcare workers and their household members provided an ideal opportunity to address the question of the effect of vaccination on transmission; transmission is commoner in households than in any other setting,[5] healthcare workers and their household members are at increased risk of COVID-19 enriching the cohort for events,[4] and healthcare workers have been prioritised for early-vaccination allowing this question to be addressed rapidly. Nevertheless, several points need to be considered when interpreting our results. First, the healthcare workers in our analysis were aged between 18 and 65 years of age. We are therefore unable to evaluate the impact on vaccinating elderly or vulnerable individuals. Secondly, differences in individual behaviour concerning testing before and after vaccination could introduce bias. However, this seems an unlikely explanation for our findings since similar results were seen for hospitalization, consistent results were obtained following the first and second dose, the differences became larger over time which is biologically plausible and the direction of effect were similar in both household members and healthcare workers.

1.3 Supplementary figures

1.3.1 Supplement figure 1: Overview of record linkage

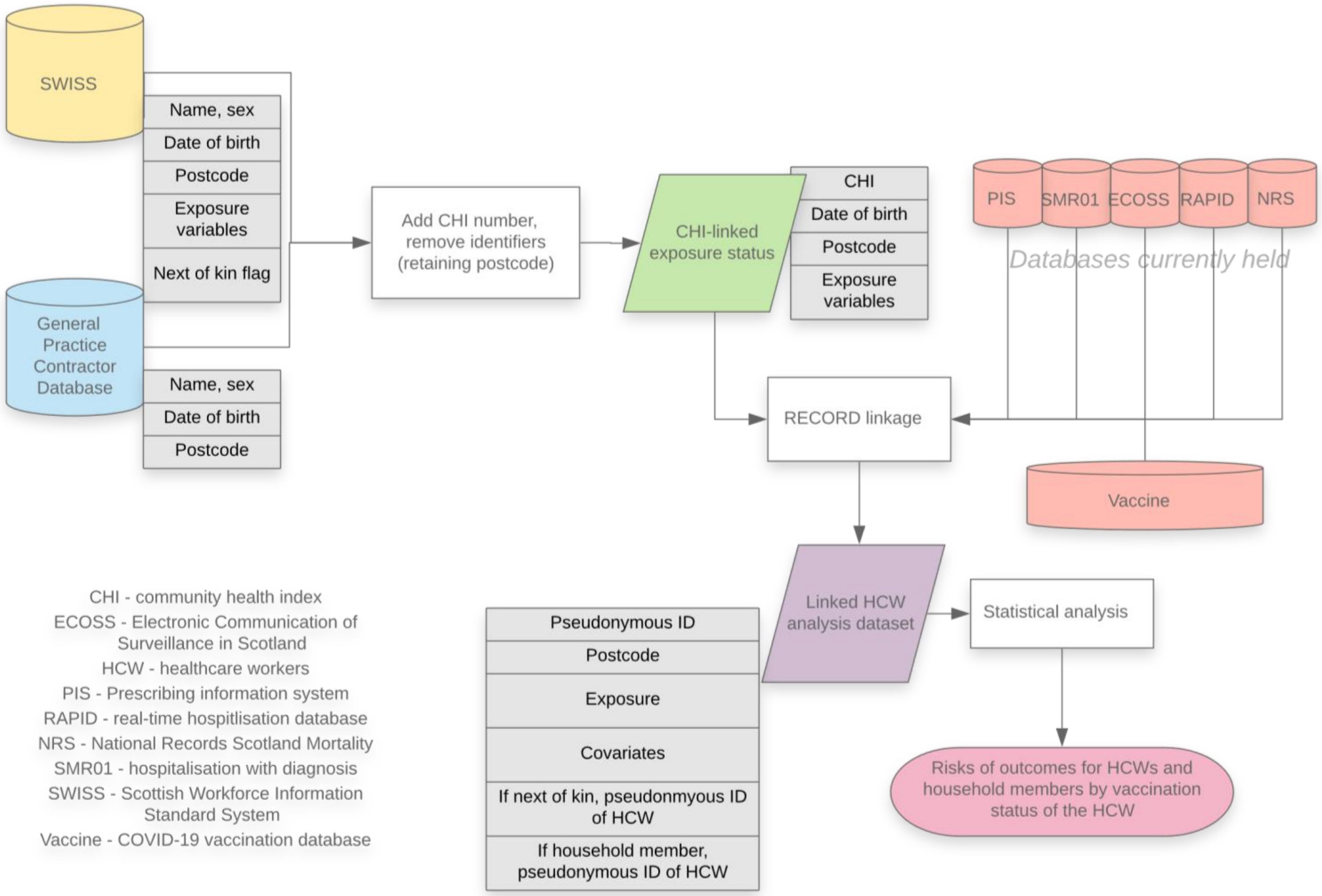

1.3.2 Supplement figure 2: Participant inclusion and exclusion for healthcare workers and their household members

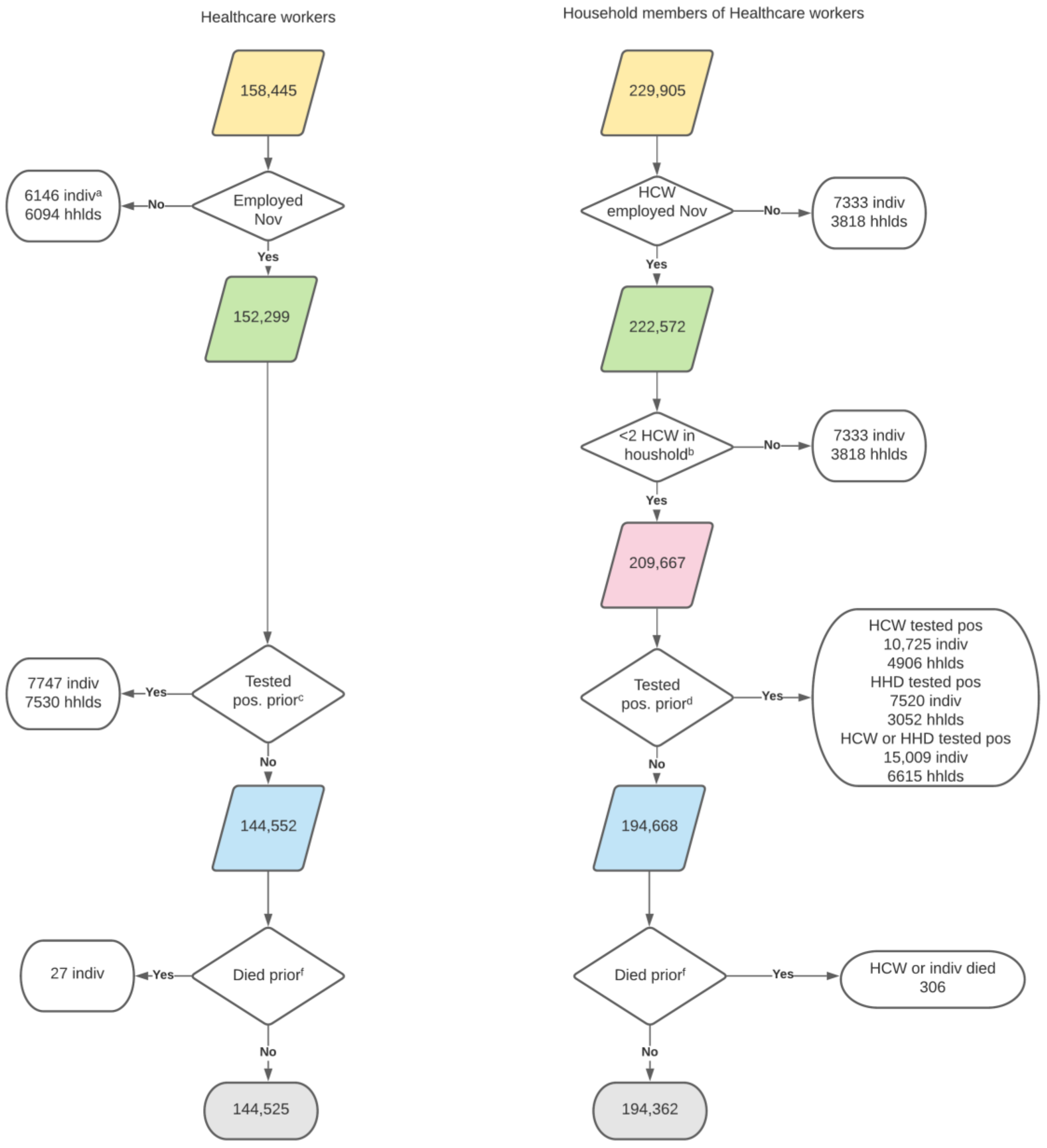

a) 4735/6146 (77.0%) were female and had a mean (standard deviation) age of 47.1 (14.4) years. 11,965 HCWs were employed from November 2020 (not included in analysis or shown on flow-chart) and had a mean (standard deviation) age of 35.5 (12.2) years and 74.7% were female (see table 1 for comparison).

b) Restriction applied solely to the household members cohort.

c) Excluded only if individual tested positive.

d) Excluded if anyone in household had tested positive (either healthcare workers or other household members).

f) Deaths on or before the 8th of December 2020.

1.3.3 Supplement figure 3: Heatmap indicating date of first and second dose of vaccination among healthcare workers. Lighter color indicates higher number receiving vaccines on those dates.

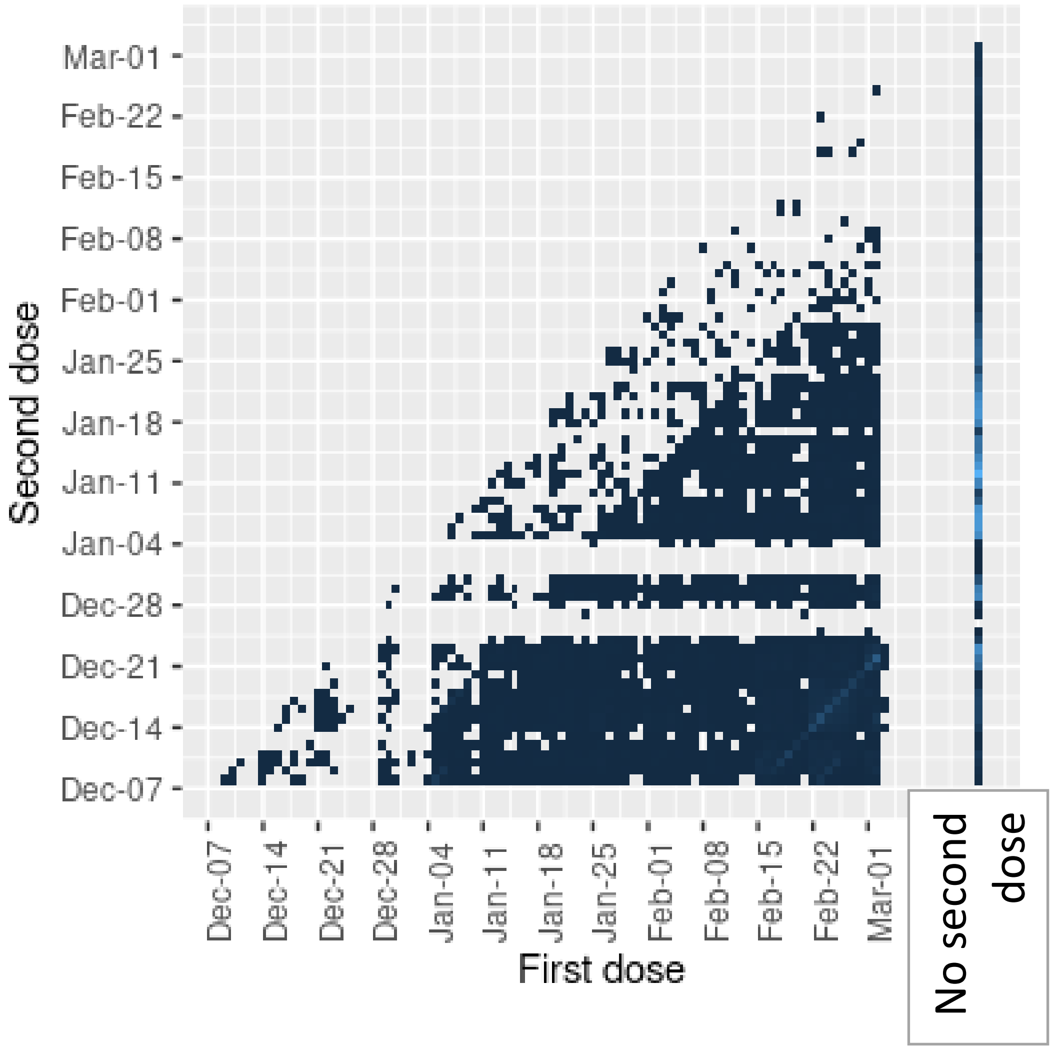

1.4 Supplement tables

1.4.1 Table 1 Baseline characteristics of vaccinated and unvaccinated and their household members by month of first dose in healthcare workers.

	**Healthcare workers**				**Household members**			
	Dec-20	Jan-21	Feb-21	Unvaccinated	Dec-20	Jan-21	Feb-21	Unvaccinated
n	49141	55618	9498	30268	65399	75787	12497	40679
Age (mean (SD)), years	44.92 (11.20)	45.30 (11.22)	46.95 (11.68)	41.28 (11.37)	31.29 (20.68)	31.41 (20.95)	32.86 (21.49)	29.74 (20.93)
Sex, male (%)	11678 (23.8)	10961 (19.7)	1933 (20.4)	6175 (20.4)	40337 (61.7)	47347 (62.5)	7837 (62.7)	24875 (61.1)
White (%)	47000 (95.6)	54364 (97.7)	9256 (97.5)	29192 (96.4)	62319 (95.3)	73347 (96.8)	11991 (96.0)	38723 (95.2)
SIMD deprivation (%)								
1 (most deprived)	5826 (11.9)	8923 (16.0)	1837 (19.3)	5161 (17.1)	6839 (10.5)	10700 (14.1)	2279 (18.2)	6328 (15.6)
2	8280 (16.8)	10766 (19.4)	2012 (21.2)	6081 (20.1)	10460 (16.0)	13995 (18.5)	2510 (20.1)	8034 (19.7)
3	9528 (19.4)	11230 (20.2)	1871 (19.7)	5886 (19.4)	12460 (19.1)	15115 (19.9)	2419 (19.4)	7805 (19.2)
4	11580 (23.6)	12538 (22.5)	2011 (21.2)	6447 (21.3)	16090 (24.6)	17812 (23.5)	2818 (22.5)	8762 (21.5)
5 (least deprived)	13927 (28.3)	12161 (21.9)	1767 (18.6)	6693 (22.1)	19550 (29.9)	18165 (24.0)	2471 (19.8)	9750 (24.0)
Role (%)[a]								
Patient facing								
Front door	19453 (39.6)	15012 (27.0)	2162 (22.8)	7012 (23.2)	26908 (41.1)	20907 (27.6)	2838 (22.7)	9760 (24.0)
Intensive care	1021 (2.1)	113 (0.2)	27 (0.3)	90 (0.3)	1101 (1.7)	117 (0.2)	19 (0.2)	93 (0.2)

	Healthcare workers				Household members			
	Dec-20	Jan-21	Feb-21	Unvaccinated	Dec-20	Jan-21	Feb-21	Unvaccinated
Other (non-AGP)	13368 (27.2)	12748 (22.9)	1608 (16.9)	5017 (16.6)	17615 (26.9)	17487 (23.1)	2168 (17.3)	6839 (16.8)
Other (AGP)	1660 (3.4)	1522 (2.7)	146 (1.5)	478 (1.6)	2353 (3.6)	2379 (3.1)	224 (1.8)	732 (1.8)
Non-patient facing	4307 (8.8)	11279 (20.3)	3376 (35.5)	11582 (38.3)	5481 (8.4)	15179 (20.0)	4422 (35.4)	15274 (37.5)
Undetermined	9332 (19.0)	14944 (26.9)	2179 (22.9)	6089 (20.1)	11941 (18.3)	19718 (26.0)	2826 (22.6)	7981 (19.6)
Comorbidity count %[b]								
None	42531 (86.5)	48330 (86.9)	7393 (77.8)	27284 (90.1)	59558 (91.1)	68798 (90.8)	11109 (88.9)	37226 (91.5)
One	5347 (10.9)	5920 (10.6)	1552 (16.3)	2467 (8.2)	4339 (6.6)	5166 (6.8)	984 (7.9)	2610 (6.4)
Two	950 (1.9)	1012 (1.8)	379 (4.0)	394 (1.3)	953 (1.5)	1111 (1.5)	236 (1.9)	539 (1.3)
Three and above	313 (0.6)	356 (0.6)	174 (1.8)	123 (0.4)	549 (0.8)	712 (0.9)	168 (1.3)	304 (0.7)
Type 2 diabetes	1174 (2.4)	1267 (2.3)	501 (5.3)	396 (1.3)	1294 (2.0)	1560 (2.1)	350 (2.8)	744 (1.8)
Vaccination data (first dose)								
ChAdOx1 nCoV-19	-	1252 (2.3)	3526 (37.1)	0 (0.0)	-	1759 (2.3)	4340 (34.7)	0 (0.0)
BNT162b2 mRNA	48564 (98.8)	54015 (97.1)	5896 (62.1)	0 (0.0)	64636 (98.8)	73529 (97.0)	8038 (64.3)	0 (0.0)
Unvaccinated	0 (0.0)	0 (0.0)	0 (0.0)	30268 (100.0)	0 (0.0)	0 (0.0)	0 (0.0)	40679 (100.0)
Unknown	577 (1.2)	351 (0.6)	76 (0.8)	0 (0.0)	763 (1.2)	499 (0.7)	119 (1.0)	0 (0.0)
Vaccination data (second dose)								

	Healthcare workers				Household members			
	Dec-20	Jan-21	Feb-21	Unvaccinated	Dec-20	Jan-21	Feb-21	Unvaccinated
ChAdOx1 nCoV-19	103 (0.2)	243 (0.4)	22 (0.2)	0 (0.0)	145 (0.2)	331 (0.4)	30 (0.2)	0 (0.0)
BNT162b2 mRNA	31983 (65.1)	3836 (6.9)	40 (0.4)	0 (0.0)	42136 (64.4)	5307 (7.0)	58 (0.5)	0 (0.0)
Unvaccinated	16978 (34.5)	51482 (92.6)	9429 (99.3)	30268 (100.0)	23020 (35.2)	70055 (92.4)	12401 (99.2)	40679 (100.0)
Unknown	77 (0.2)	57 (0.1)	7 (0.1)	0 (0.0)	98 (0.1)	94 (0.1)	8 (0.1)	0 (0.0)

AGP - aerosol generating procedure

[a] We defined roles on the basis of formal job titles for nursing staff, allied health professionals, and support staff and specialty for medical staff. Selected nursing staff were additionally assigned on the basis of their working location (for example, the emergency department). We deliberately made these definitions narrow, assigning around a fifth of healthcare workers to “undetermined”. We did this to avoid non-differential misclassification bias. We further divided patient facing roles into the following settings: “front door” (for example, paramedics or workers in acute receiving specialties), intensive care, non-intensive care but still exposed to aerosol generating procedures (for example, workers in respiratory medicine), and “other.” A list of job titles and assignment of roles is already published (Shah et al, BMJ 2020;371:m3582)

[b] Comorbidity was defined using ICD-10 codes. While the accuracy of ICD 10 coding in Scotland remains high and can be accessed at https://beta.isdscotland.org/products-and-services/data-quality-assurance/dqa-assessments/ for sample conditions

1.4.2 Table 2: Estimation of the number of cases prevented in household members by vaccinating healthcare workers as a fraction of the number of cases preventable via interrupting this route of transmission (ie those cases caused by sharing a household with a healthcare worker)

Effect of sharing a household with a healthcare worker (HCW)		Effect of vaccination on all detectable cases		Effect of vaccination on preventable cases
Relative risk RR_{HHD}	Attributable fraction among the exposed $AF_{HHD)} = (RR_{HHD} -1)/ RR_{HHD}$	Relative risk (RR_{vax})	Relative risk reduction $RRR_{vax} = 1- RR_{vax}$	Proportion of cases prevented = RRR_{vax}/AF_{HHD}
1.5	33.3%[$]	0.70 (0.63 to 0.78)	30% (22-37)	90% (66 to 111)[$]
**2.0***	**50.0%**	**0.70 (0.63 to 0.78)**	**30% (22-37)**	**60% (44 to 74)**
2.5	60.0%	0.70 (0.63 to 0.78)	30% (22-37)	50% (37 to 62)
3.0	66.7%	0.70 (0.63 to 0.78)	30% (22-37)	45% (33 to 55)

This tables shows the results of an illustrative post hoc analysis of the impact of vaccinating healthcare workers (HCWs) on transmission, taking into account that only a proportion of cases in household members could plausibly be prevented by vaccination HCWs, since they can also be infected via other routes.

The degree of infection that occurs via other routes will vary. During tight societal lockdown the majority of cases in household members will come via the healthcare worker. When societal restrictions are lifted, the opposite may be true. The relative risk from living with a healthcare worker will therefore range from large values (as seen early in the pandemic) towards one (when societies are least restricted). For this reason, it is not possible to produce a single estimate for the effect of vaccination on transmission via the healthcare worker-household member link.

As such, we show a range of plausible effects under different plausible estimates for the causal effect of sharing a household with a healthcare worker on the risk of developing COVID-19. The estimate in the second row (RR_{HHD} = 2.0) is close to our own previous empirical estimates for this association (BMJ 2020;371:m3582).

To perform thus calculation we made the following assumptions:-

- Vaccination of HCWs can only reduce the risk in household members by interrupting transmission from HCWs to household members and not via other mechanisms (eg by causing a modification of behavior in household members)
- That the estimated association for vaccination is a valid causal estimate – ie no bias and no confounding

*The estimate in the second row (RR_{HHD} = 2.0) is close to our own previous empirical estimates for this association (BMJ 2020;371:m3582).

[$] Since the upper limit is over 100% (column 5), the upper bound of our 95% CI effect estimate (column 4) is not compatible with 33.3% (column 2) of cases in household members being caused by exposure to healthcare workers.

1.4.3 Table 3: Effect of vaccination in healthcare workers on documented COVID-19 in healthcare workers and their household at multiple time periods following the first dose

			Days from vaccination				
Population	**Outcome**	**Unvaccinated period**	**Day 1-6**	**Day 7-13**	**Day 14-20**	**Day 20-27**	**Post day 28**
Healthcare workers	Cases	1	0.81 (0.72 to 0.90)	1.11 (1.02 to 1.22)	0.54 (0.47 to 0.61)	0.42 (0.36 to 0.49)	0.43 (0.39 to 0.47)
Healthcare workers	Hospitalisation	1	0.48 (0.25 to 0.92)	0.78 (0.48 to 1.27)	0.16 (0.06 to 0.43)	0.13 (0.04 to 0.40)	0.17 (0.09 to 0.32)
Household	Cases	1	0.73 (0.61 to 0.87)	1.08 (0.94 to 1.25)	0.85 (0.73 to 0.99)	0.68 (0.58 to 0.81)	0.64 (0.56 to 0.73)
Household	Hospitalisation	1	0.95 (0.53 to 1.69)	1.25 (0.78 to 2.02)	0.96 (0.56 to 1.63)	0.83 (0.47 to 1.46)	0.64 (0.40 to 1.01)

Results shown are hazard ratios from Cox models adjusting for the variables shown in the footnote of Table 2 (model 4). Caution is needed when interpreting the effect of vaccination on hospitalization with COVID-19 given the wide confidence intervals.

1.4.4 Table 4: Effect of second dose vaccination in healthcare workers on documented COVID-19 cases and hospitalizations in healthcare workers and their households: unvaccinated period versus periods from 14-days post second dose

	**Healthcare workers**				**Household members of healthcare workers**			
	Cases		Hospitalizations		Cases		Hospitalizations	
	Unvaccinated	Post second dose	Unvaccinated	Post second dose	Unvaccinated	Post second dose	Unvaccinated	Post second dose
n	144525	12592	144525	12725	194362	16895	194362	17045
Events	3191	8	158	0	2037	27	111	2
Mean person time (days)	40.03	19.78	40.96	19.75	40.67	19.56	41.05	19.55
Rate per 100 person years	20.13	1.17	0.97	0.00	9.40	2.98	0.51	0.22
Models (Hazard ratios)								
Unadjusted	0.10 (0.05-0.19)		-		0.48 (0.32-0.73)		0.71 (0.17-2.92)	
Model 1	0.10 (0.05-0.20)		-		0.47 (0.31-0.72)		0.68 (0.17-2.84)	
Model 2	0.11 (0.05-0.22)		-		0.50 (0.33-0.76)		0.78 (0.19-3.24)	
Model 3	0.08 (0.04-0.17)		-		0.46 (0.30-0.70)		0.69 (0.17-2.83)	
Model 4	0.08 (0.04-0.17)		-		0.46 (0.30-0.70)		0.68 (0.17-2.83)	

Results shown are hazard ratios from Cox models adjusting for the variables shown in the foot of the primary published table (model 4). Caution is needed when interpreting the effect of vaccination on hospitalization with COVID-19 given the wide confidence intervals.

1.5 References

1. Bennie M, Malcolm W, Marwick CA, Kavanagh K, Sneddon J, Nathwani D. Building a national Infection Intelligence Platform to improve antimicrobial stewardship and drive better patient outcomes: the Scottish experience. *J Antimicrob Chemother*. 2017 Oct 1;72(10):2938-42.
2. NHS National Services Scotland (NSS), 2014. Infection Intelligence Platform (IIP)- High level guide to IIP component datasets held by NHS National Services Scotland, https://www.isdscotland.org/Health-Topics/Health-and-Social-Community-Care/Infection-Intelligence-Platform/Data/June-2014-Guide-to-IIP-Data.pdf (accessed 18th March, 2020)
3. Scottish Government, 2014. Scottish Management of Antimicrobial Resistance Action Plan 2014-2018. http://www.gov.scot/Publications/2014/07/9192 (accessed 2nd March, 2020)
4. Shah ASV, Wood R, Gribben C, et al. Risk of hospital admission with coronavirus disease 2019 in healthcare workers and their households: nationwide linkage cohort study. BMJ 2020;371:m3582.
5. Madewell ZJ, Yang Y, Longini IM, Jr., Halloran ME, Dean NE. Household Transmission of SARS-CoV-2: A Systematic Review and Meta-analysis. JAMA Netw Open 2020;3:e2031756.

1.6 Study protocol

Public Health Scotland

Protocol for Study of Vaccine Efficacy in Health Care Workers

[Document subtitle]

Anoop Shah, Peter Hanlon, Paul M McKeigue, Helen M Colhoun, David McAllister

3-3-2021

Table of Contents

Abbreviations 1
Responsible parties 2
Abstract 2
Amendments and updates 2
Milestones and timeline 2
Rationale and Background 2
Research question and objectives 3
Research methods 3
Study design 3
Feasibility stage 3
Vaccine effectiveness estimation stage 3
Setting 3
Variables 3
Exposure assessment and time at risk 3
Eligibility criteria 4
Outcome assessment 4
Covariate assessment 4
Data sources 5
Study size 5
Data management 5
Data analysis 5
Subgroup, sensitivity and exploratory analyses 6
Missing data 6
Quality control 6
Limitations of the research methods 6
Protection of human subjects 7
Management and reporting of adverse events/adverse reactions 7
Plans for disseminating and communicating study results 7
Other good research practice 7

Abbreviations

HCW: Healthcare worker

Responsible parties

Public Health Scotland staff and honorary staff.

Abstract

Background

Decisions around COVID-19 vaccination strategies and non-pharmacological interventions require evidence on the effectiveness of vaccines in protecting individuals vaccinated from infection and severe disease, but also the impact of vaccination on transmission, in real-world contexts.

Objectives

1. To determine the real-world efficacy of COVID-19 vaccination in HCWs.
2. To determine the effect of vaccinating HCWs on the risk of COVID-19 in individuals with whom they share a household as such an effect would be consistent with an effect of vaccination on transmission

Methods

Record linkage of routine healthcare data.

Amendments and updates

This is the first version of the protocol submitted to ENCEPP. As the ENCEPP server was unavailable when we ready to start the analysis we posted an encrypted version of an SAP on a public github repository (https://github.com/dmcalli2/hcw_vax). We have followed closely the ACCESS template created in ENCEPP.

Milestones and timeline

This document was created after linking the vaccination data to the healthcare worker/household cohorts but BEFORE these data were linked to the outcome data (except outcomes prior to July 2020 which were already linked). Where this description refers to the “original” analyses this corresponds to the analysis of COVID-19 risk in healthcare workers (HCWs) carried out for the first wave of the pandemic and described in https://www.bmj.com/content/371/bmj.m3582.

Rationale and Background

Decisions around COVID-19 vaccination strategies and non-pharmacological interventions require evidence on the effectiveness of vaccines in protecting individuals vaccinated from infection and severe disease, but also the impact of vaccination on transmission, in real-world contexts.

Healthcare workers and their household members provide an ideal population to address these question since: -

1. Healthcare workers are essential to providing health services and so their health is of wider public interest
2. Healthcare workers are a high risk population so the number of events are likely to be higher
3. Healthcare workers have been vaccinated early, allowing us to examine the questions at the earliest possible opportunity
4. Individuals who are household members of healthcare workers are at increased risk compared to people who are not household members of healthcare workers. As such

studying the impact of vaccinating healthcare workers on their household members is a good proxy for measuring transmission.

Research question and objectives

3. To determine the real-world efficacy of COVID-19 vaccination in HCWs.
4. To determine the effect of vaccinating HCWs on the risk of COVID-19 in individuals with whom they share a household as such an effect would be consistent with an effect of vaccination on transmission.

Research methods

Study design

Feasibility stage

This study will use a previously defined cohort of healthcare workers in Scotland and their household members, with existing linkage to demographic data, health-associated outcomes, and virology data for SARS-CoV-2 status. This cohort has previously been used to assess the impact of COVID-19 on healthcare workers (https://www.bmj.com/content/371/bmj.m3582). Linkage will be updated to include vaccination status and updated outcomes in the post-vaccination period.

Vaccine effectiveness estimation stage

Due to lack of availability we do not have a negative control outcome analysis. The protocol that follows describes the vaccine effectiveness estimation stage. Possible residual confounding is discussed under the limitation section below.

Setting

This is a national-level study of data from Scotland.

This study is based on healthcare workers and their households in Scotland. Data for healthcare workers are from the Scottish Workforce Information Standard System (SWISS) and General Practitioner Contractor Database (GPCD). These data were linked to national databases using the Community Health Index (CHI) database. The CHI database is a registry of all patients registered to receive National Health Service (NHS) care in Scotland.

People who are not healthcare workers but share a household with a healthcare worker were identified using the CHI database. Therefore this is a national-level cohort based on the cohort of healthcare workers. Full details of the linkage used to define the cohort are given here: https://www.bmj.com/content/371/bmj.m3582

Variables

Exposure assessment and time at risk

For healthcare workers:

At least one dose of any COVID-19 vaccine. Currently those in use in Scotland are Vaccination are the BNT162b2 mRNA COVID-19 (also known as Pfizer BioNTech) and the ChAdOx1 nCoV-19 (AZD1222) Oxford-AZ vaccine).

For non-healthcare worker household members:

At least one dose of any COVID-19 vaccine (BNT162b2 mRNA COVID-19 and the ChAdOx1 nCoV-19 (AZD1222)) in a HCW with whom a non-healthcare worker shares a household.

Study period

The study period will be from the 8th of December 2020 to the 21st of February 2021. Those eligible for entry to the study will contribute person time throughout this period. Person time will be right censored on date of event (eg first positive test), or death from non-COVID-19 cause or study end date.

Eligibility criteria

Healthcare workers:

HCWs (patient-facing, non-patient facing or undetermined) who meet the following criteria:-

- Employed on or before March 2020 (and included in the cohort described here https://www.bmj.com/content/371/bmj.m3582)
- Still in employment as a HCW in November 2020
- No positive PCR test for COVID-19 on or before the 8th of December 2020 (the date of the first vaccine)
- Aged 21 to 65 years

Non-healthcare worker household members:

Household members of HCWs (patient-facing, non-patient facing or undetermined) who are not themselves HCWS, where a single HCW in the household meets the above criteria.

The analysis of household members will be restricted to households where there is no more than one HCW (this covers approx. 95% of household members) to avoid complexity. HCWs themselves are not included in this analysis. It will also exclude all households where any household member tested positive for SARS-CoV-2 on or before the 8th of December 2020. Note that household members are not restricted to specific ages.

Outcome assessment

Primary outcome

Any case of COVID-19 defined as the first positive test for SARS-CoV-2.

Secondary outcomes

Hospitalisation with COVID-19 defined as the first positive test for SARS-CoV-2 in hospital and/or the individual being admitted within 28 days of testing positive, and/or death or hospitalisation with COVID-19 recorded as the cause (ICD-10 codes U07.1 or U07.2). Where an individual is tested this will be taken as the date of the event. Where hospitalisation or death occur without any positive test, infection will be presumed to have occurred 7 days prior to the date of hospitalisation and/or death.

Severe COVID-19, ie COVID-19 resulting in ICU admission or death, defined as the first admission to intensive care or death within 28 days of testing positive for SARS-CoV-2, and/or death with COVID-19 recorded as the cause (ICD-10 codes U07.1 or U07.2). Where ICU admission or death occur without any positive test, infection will be presumed to have occurred 7 days prior to the date of hospitalisation and/or death.

Covariate assessment

- age (using a penalised spline to allow for non-linearity),
- sex,
- Scottish Index of Multiple Deprivation (SIMD),
- ethnicity (white versus other),
- comorbidity (as both a comorbidity count and the presence/absence of type 2 diabetes),

- role (patient facing, non-patient facing, undetermined),
- occupation (any of nursing and midwifery, medical and dental or allied health profession versus other), and part-time status (whole time versus part-time).

The comorbidity count will be based on the following comorbidities; ischaemic heart disease, other heart disease, other circulatory system diseases, asthma and chronic lower respiratory disease, neurological disorders, decompensated liver disease, any immune disorder, malignant neoplasms, disorders of oesophagus, stomach and duodenum, type 1 diabetes, type 2 diabetes, and diabetes of unknown type. The definition of each of these covariates has previously been described (https://www.bmj.com/content/371/bmj.m3582).

Data sources

Data for healthcare workers are from the Scottish Workforce Information Standard System (SWISS) and General Practitioner Contractor Database (GPCD). These data were linked to national databases using the Community Health Index (CHI) database.

Vaccination status for healthcare workers will be ascertained through linkage to the Public Health Scotland/NHS Education Scotland vaccine database.

CHI database was used to link both healthcare workers and their households to multiple national-level databases including hospital admission data, community prescriptions, critical care admissions, national register for deaths, and virology testing for SARS-CoV-2.

This linkage is already in place and has been used for a previous analysis of COVID-19 outcomes in healthcare workers (https://www.bmj.com/content/371/bmj.m3582).

Study size

The healthcare worker and household cohort has previously been described https://www.bmj.com/content/371/bmj.m3582.

Data management

Data will be managed within Public Health Scotland, who hold these data, according to the organisation's internal policies.

Data analysis

Healthcare worker analysis

The model will compare the rates of the events in person time exposed to vaccine versus unexposed to vaccine as hazard ratios. Extended Cox regression models will be fitted with vaccination treated as a time-varying covariate. 4% of HCWs live in multiple HCW households. Robust standard errors will therefore be estimated to allow for clustering due to shared household membership (cluster function in survival::coxph). The same groups of health board areas will be used to stratify the analysis as per the original analysis of COVID-19 risk in HCWs and household members to allow for differences in baseline hazard between geographic areas.

From the model we will report the HR associated with vaccination status at 7-day intervals from the date of vaccination. However, note that the primary efficacy parameter will be the HR for events in the period 14 days onward from vaccination compared to un–vaccinated person time (ie before day 1 of vaccination).

Factors identified as potential risk factors for COVID-19 hospitalisation in the original analysis will be included as covariates in the full model for the primary analysis – age (using a penalised spline to

allow for non-linearity), sex, Scottish Index of Multiple Deprivation (SIMD), ethnicity (white versus other), comorbidity (as both a comorbidity count and the presence/absence of type 2 diabetes), role (patient facing, non-patient facing, undetermined), occupation (any of nursing and midwifery, medical and dental or allied health profession versus other), and part-time status (whole time versus part-time). The comorbidity count will be based on the following comorbidities; ischaemic heart disease, other heart disease, other circulatory system diseases, asthma and chronic lower respiratory disease, neurological disorders, decompensated liver disease, any immune disorder, malignant neoplasms, disorders of oesophagus, stomach and duodenum, type 1 diabetes, type 2 diabetes, and diabetes of unknown type. The definition of each of these covariates has previously been described (https://www.bmj.com/content/371/bmj.m3582). For transparency, a minimally adjusted model will also be presented adjusting solely for age, sex and SIMD, as will intermediate models between the minimal and the full model.

Non-healthcare worker household member analysis:
The model will compare the rates of the events in person time exposed to a vaccinated HCW who is member of household versus person time exposed to an unvaccinated HCW who is member of household as hazard ratios. Extended Cox regression models will be fitted with the vaccination of the HCW treated as a time-varying covariate. Robust standard errors will be estimated to allow for clustering due to shared household membership (cluster function in survival::coxph). The same stratifying variable will be used as in the healthcare worker analysis

From the model we will report the HR associated with vaccination status of the HCW in the household at 7-day intervals from the date of vaccination however note that the primary efficacy parameter will be the HR for events in the period 14 days onward from vaccination compared to un–vaccinated person time (ie before day 1 of vaccination). For simplicity, we opted to use the same periods of comparison as the HCW analysis.

The same covariates will be modelled as in the healthcare worker analysis, except that occupation-related variables (occupation, role, part-time status) will refer to the HCW with whom the household member shares a household, not the household member themselves.

Subgroup, sensitivity and exploratory analyses

Missing data
Imputation for ethnicity and part-time status will be carried out as per the original analysis.

Quality control
All analyses will be conducted using scripting languages under version control software. Code will be independently reviewed by a second analyst. Outputs will be created using literate programming software. Data will be stored, backed-up and archived according to standard Public Health Scotland policies.

Limitations of the research methods
It is possible that household members of HCWs with COVID-19 may be less likely to present for a test when they know that the HCW with whom they live has been vaccinated. However, such an effect would depend upon symptomatic household members assuming that they could not have been exposed to SARS-COV-2 via any other route than via the HCW with whom they live. It is not plausible that this will be a large effect.

Protection of human subjects

This is a non-interventional study using secondary data collection and does not pose any risks for patients. Approval for data access and linkage has been granted by the Scottish Public Benefit and Privacy Panel for Health and Social care data. Data protection and privacy regulations will be observed in collecting, forwarding, processing, and storing data from study participants.

Management and reporting of adverse events/adverse reactions

Any unexpected increase in risk identified will be detailed within the study report, in accordance with the International Society for Pharmacoepidemiology (ISPE) *Guidelines for Good Pharmacoepidemiology Practices (GPP*).

Plans for disseminating and communicating study results

Findings will be disseminated through submission to a peer-reviewed journal.

Other good research practice

This study will adhere to the Guidelines for Good Pharmacoepidemiology Practices (GPP) and has been designed in line with the European Network of Centres for Pharmacoepidemiology and Pharmacovigilance (ENCePP) Guide on Methodological Standards in Pharmacoepidemiology. The ENCePP Checklist for Study Protocols will be completed.
The study is a postauthorisation study of vaccine effectiveness and will comply with the definition of the non-interventional (observational) study referred to in the International Conference on Harmonisation tripartite guideline Pharmacovigilance Planning E2E and provided in the EMA Guideline on Good Pharmacovigilance Practices (GVP) Module VIII: Post-Authorisation Safety Studies, and with the 2012 European Union pharmacovigilance legislation, adopted 19 June 2019.
The study will comply with the study reporting requirements specified in Module VIII section VIII.B.6.3.1. “Progress reports” and VIII.B.6.3.2. “Final study report” of the Guideline of Good Pharmacovigilance Practices.
The study will be registered in the EU PAS Register before study implementation commences.
